# Analysis of population‐based colorectal cancer screening in Guangzhou, 2011‐2015

**DOI:** 10.1002/cam4.1867

**Published:** 2019-03-29

**Authors:** Feng Zhiqiang, Cao Jie, Nie Yuqiang, Gong Chenghua, Wang Hong, Sun Zheng, Li Wanglin, Zhou Yongjian, Dai Liping, Zeng Lizhong, Zhao DeJian

**Affiliations:** ^1^ Department of Gastroenterology Guangzhou First People's Hospital, The Second Affiliated Hospital of South China University of Technology Guangzhou China; ^2^ Department of Gastrointestinal Surgery Guangzhou First People's Hospital, The Second Affiliated Hospital of South China University of Technology Guangzhou China; ^3^ Yuexiu District Center for Disease Control and Prevention Guangzhou China

**Keywords:** advanced adenomas, colorectal cancer, Guangzhou, polypus, screening

## Abstract

**Objective:**

To analyze the detection rates of colorectal cancer (CRC) and polyps by population‐based screening in Guangzhou.

**Methods:**

From January 2011 to December 2015, the residents aged 30‐79 were selected for CRC screening. The residents were conducted Questionnaires and/or FOBT to assess high‐risk groups, the free colonoscopy examination was recommended, and the results were evaluated in detail.

**Results:**

There were 98 927 residents involving screening, 5306 high‐risk residents identified (males 1859 and females 3447), and 4713 subjects underwent colonoscopy (males 1690 and females 3023). CRC was seen in 55 individuals (males 28 and females 27), and the detection rates in male were higher than in female (*P* = 0.019). And the detection rates increasing with age, for people over 60 years old, were obviously higher than those younger (*x* ^2^ = 18.64, *P* = 0.000924).

The polyps were seen in 1458 (30.94%) cases， and 1420 subjects received pathological examination (adenomas 971 and non‐adenomatous polyps 449). Advanced adenomas were seen in 462 cases (males 240 and females 222) and 509 cases of non‐advanced adenomas (males 255 and females 254). For advanced adenomas, the detection rates in male were higher than female (14.20% vs 7.34%, *P* = 2.64 × 10^−14^). For the detection rates of adenomas or advanced adenomas by age, the people over 40 years were higher than younger (20.91% vs 3.61% *P* = 7.87 × 10^−6^; 9.94% vs 2.41%, *P* = 0.009).

**Conclusions:**

For Guangzhou residents, the detection rates of CRC and adenoma were 1.17% and 20.60%. The detection rates of CRC increasing with age, for people over 60 years old, were obviously higher than those younger. But for people over 40 years, the detection rate of adenoma and advanced adenoma was higher than younger. So for people over 40 years, the CRC screening is recommended.

## 1 INTRODUCTION

With the social and economic development and the westernization lifestyle and diet, the incidence rates of colorectal cancer were significantly increasing in China.

In 2010, the crude CRC incidence rate in China was 20.1/100 000,^1^ while in 2011, the crude incidence rate was 23.03/100 000.^2^ In Guangzhou, CRC crude incidence increased from 22.2/10^5^ in 2000 to 34.0/10^5^ in 2011,^3^ higher than the average of China for the same time.

The majority of CRC cases (>95%) developed from a preclinical precursor, the adenomatous polyps,^4^, ^5^ which arising from aberrant proliferation of epithelial cells in the colon. These lesions may then progress to varying degrees in size and dysplasia,^6^ and the progression from early adenoma to invasive cancer takes some years.^7^, ^8^ Approximately 0.25% of adenomas will progress to cancer per year.^9^

Advanced adenoma was defined with adenomas possessing at least one of three high‐risk characteristics: (1) size at least 10 mm in greatest diameter, (2) exhibiting advanced histology such as villous, tubulovillous of at least 25% with villous features, (3) high‐grade dysplasia.^8^, ^10^, ^11^, ^12^

For people over 55 years old, the estimated annual incidence rate of non‐advanced adenomas ranged from 1.4% to 2.4%,^13^ annual transitions from non‐advanced to advanced adenoma were about 3.6% to 4.7%, cumulative transition rates are expected to be close to 30%,^14^ the estimated annual transition rates from advanced adenoma to CRC were 2.4% to 6.3%, and the cumulative transition rates are expected to exceed 40%.^14^

In the United States in 2017, there are projected to be 135 430 individuals newly diagnosed with CRC, of which 45% of men and 39% of women are younger than the age of 65 years at diagnosis, and 11% of men and 10% of women are under 50 years at diagnosis.^15^ Bailey et al predicted that by 2030, the incidence of CRC would increase by 27.7% to 46.0% in individuals aged 35 to 49 years.^16^

Compared with general population, First‐degree relatives of patients with CRC are at higher risk of developing CRC.^17^, ^18^ So for first‐degree relative with CRC, colonoscopic screening is recommended from the age of 40 or 10 years before the youngest case in the immediate family.^18^, ^19^, ^20^ For those reasons, we select residents aged 30‐80 years old as screening population.

From the latest two decades, the incidence rates and mortality rates of CRC declined in the United States, and the cumulative effect of CRC screening strategies is responsible for 50% of the decline in incidence and mortality rates of CRC in the United States.^21^ We started CRC screening from November 2010 in Yuexiu District, Guangzhou, and achieved good results under the supporting of special funds provided by Ministry of Health.

## 2 MATERIALS AND METHODS

### 2.1 The study population and methods

FOBT was widely used in CRC screening, but has a less perfect sensitivity, so some scholars established risk factors for CRC screening, and used Harvard Cancer Risk Index to identify high‐risk factors of cancer^22^, ^23^, ^24^, ^25^; here, we conducted FOBT combined with other risk factors to select high‐risk people in our preliminary CRC screening stage.

The community residents aged 30‐80 years old in Yuexiu District, Guangzhou，were selected as preliminary screening population from January 2011 to December 2015. A 2‐stage colorectal cancer screening program was conducted. For the first stage, the community residents were conducted Questionnaires and/or FOBT to assess high‐risk groups. The enrolled participants complete a Questionnaires, including the following: (a) For the following symptoms, there were at least two positive: chronic constipation, chronic diarrhea, mucus bloody stool, chronic appendicitis or appendectomy, chronic biliary tract disease, or cholecystectomy; (b) for every resident, FOBT were conducted twice by two different stool samples, and at least one positive; (c) the history of colonic polyps or CRC; (d) first‐degree relatives with colorectal cancer; and (e) first‐degree relatives of familial adenomatous polyposis. Patients with severe other diseases, such as severe heart disease, severe brain disease, severe lung disease, severe liver disease, severe kidney disease, and severe mental illness, were excluded. For every participating resident, the FOBT positive or at least one of the five Questionnaires positive was defined as high‐risk people.

The above information was reviewed and collected by trained medical personnel. All data were inputted into a database with unique Resident Identity Card numbers. For every participating resident, the unique Resident Identity Card numbers were inputted into a database. For those high‐risk people, the free colonoscopy examination was recommended. The results of colorectal screening were evaluated in detail. The retrospective analysis protocol was approved by the Ethics Committee of Guangzhou First People's Hospital.

### 2.2 FOBT reagent

The FOBT reagents are purchased from WHPM Bioresearch and Technology Co., Ltd, which is One‐step Immunological Hemoglobin Test, the cutoff point is 0.2 µg hemoglobin/mL.

### 2.3 Free colonoscopy examinations

For all high‐risk people, the free colonoscopy examinations were recommended. Orally administered mannitol was used for colonic preparation before operations. For the defects, the size, number, polyp morphology, and the anatomical position were recorded. All colonoscopy examinations were conducted by the specialist endoscopists. The colonoscopies were performed in our two hospitals (Guangzhou First People' Hospital and Sun Yat‐University Cancer Center). All data were submitted to Yuexiu District Center for Disease Control and Prevention.

### 2.4 Quality control of colonoscopy

According to different guidelines, we selected the following key quality indicators in our colonoscopy screening: (a) informed consent; (b) rate of adequate bowel preparation ≥90%; (c) cecal intubation rate ≥90%; (d) adenoma detection rate ≥25%; (e) appropriate polypectomy technique ≥80%; (f) the average withdrawal time ≥8 minutes; and (g) serious adverse event, such as perforation or bleeding, must be recorded.^26^, ^27^, ^28^, ^29^, ^30^, ^31^

### 2.5 Pathological examinations

For lesions, the biopsy was taken and the pathology examinations were carried out.

### 2.6 Statistical analyses

For multiple neoplasms, the most advanced one was recorded. Data were analyzed using the Statistical Package for Social Sciences (SPSS) software (version 10.0; SPSS, Inc, Chicago, IL, USA). The chi‐squared were used for categorical data. The statistical significance threshold was defined as *P* < 0.05.

## 3 RESULTS

From January 2011 to December 2015, a total of 98 927 participants entered population‐based colorectal cancer screening, 5306 (5.36%) were identified as high risk (1859 males and 3447 females), and 4713 (88.82％, 4713/5306) subjects underwent colonoscopy examinations, including 1690 males and 3023 females, from 30 to 79 (56.8 ± 8.5) years old. The cecum intubation rate was 97.54% (4597 of 4713), in this study, there was no perforation or no bleeding occurred.

**Figure 1:**
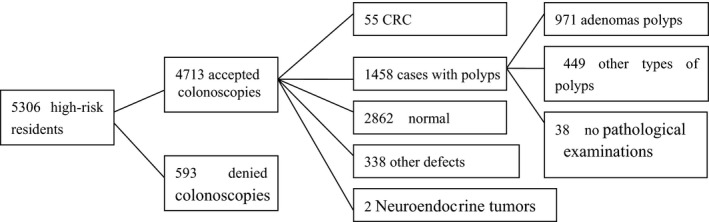
The results of the colonoscopy examinations

Totally, 55 cases (1.17%) had CRC and 1458 (30.94%) subjects had colorectal polyps. (Figure 1, Table 1).

**Table 1:** The general results of the colonoscopy examinations for gender

Results of colonoscopy	Cases of colonoscopy	Male	Female	*x* ^2^	*P* values
Normal	2862	855	2007		
CRC	55	28	27	5.48	0.019
Advanced adenomas	462	240	222	57.65	3.13 × 10^−14^
Non‐advanced adenomas	509	256	253	51.71	6.45 × 10^−13^
Neuroendocrine tumors	2	0	2		
Non‐adenomas polyps	447	189	258		
No pathological	38	16	22		
IBD	4	3	1		
Inflammation and others	334	103	231		
Total	4713	1690	3023		

The CRC detection rates were higher in male than in female (1.66%, 28/1690% vs 0.89%, 27/3023, *P* = 0.019). And the detection rates increasing with age, for people aged 40 to 49, 50 to 59, 60 to 69, and 70 to 79 years old, the detection rate was 0.61%, 0.76%, 1.69%, and 3.03%, respectively (*x* ^2^ = 18.64, *P* = 0.000924; Table 2).

**Table 2:** The numbers of CRC at different age‐groups

Age‐groups	Cases of colonoscopies	Cases of CRC	cases of none‐CRC	*x* ^2^	*P* values
30‐39	83	0	83	18.64	0.000924
40‐49	817	5 (0.61%)	812		
50‐59	1979	15 (0.76%)	1964		
60‐69	1537	26 (1.69%)	1511		
70‐79	297	9 (3.03%)	288		

The anatomical distribution of CRC as follows: ascending colon and cecum, 7 (12.73%); hepatic flexure, 2 (3.63%); transverse colon, 5 (9.09%); splenic flexure, 2 (3.63%); descending colon, 5 (9.09%); sigmoid colon, 18 (32.73%); rectal, 16 (29.10%), respectively. Taken together, there were 25.45% (14 cases) located in the right‐sided colon, 45.45% (25 cases) located in the left‐sided colon, and 29.09% (16 cases) located in rectal (Table 3).

**Table 3:** The anatomical distribution of CRC and advanced adenomas

	CRC	Advanced adenomas
Ascending colon and cecum,	7 (12.73%)	83 (17.97%)
Hepatic flexure and transverse colon	7 (12.73%)	83 (17.97%)
Splenic flexure and descending colon	7 (12.73%)	69 (14.94%)
Sigmoid colon	18 (32.72%)	156 (33.77%)
Rectal	16 (29.09%)	71 (15.37%)
Total	55 (100%)	462 (100%)

**Figure 2:**
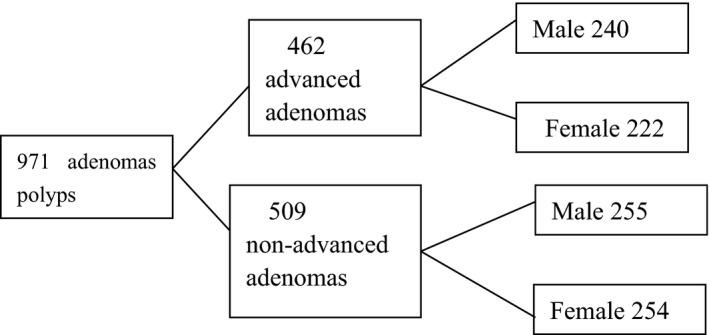
The results of the polyps

For 1458 subjects with colorectal polyps, 38 cases had no pathological examinations, 1420 received pathological examination, and 971 cases were adenomas (males 495 and females 476), including 462 cases of advanced adenomas (males 240 and females 222) and 509 cases of non‐advanced adenomas (males 255 and females 254; Figure 2, Table 1). The detection rates of advanced adenomas were higher in male (14.20%, 240/1690) than in female (7.34%, 222/3023; *P* < 0.001).

Because the adenomas progress to invasive cancer takes some years, we analyze the detection rates of adenomas and advanced adenomas at different age‐groups, and found the detection rates of adenomas and advanced adenomas increasing with age. For people over 40, 45, and 50 years old, the detection rate of adenoma was 20.91%, 21.75%, and 23.03%, respectively. Similarly, the detection rates of advanced adenomas for people over 40, 45, and 50 years old were 9.94%, 10.43%, and 11.96%, respectively, and the difference was obvious (Tables 4 and 5).

**Table 4:** The numbers of adenomas at different age‐groups

	Age‐groups	Cases of colonoscopy	Cases of adenomas	*x* ^2^	*P* values
Age‐group 1	30‐39	83	3 (3.61%)	14.906	7.87 × 10^−6^
	40‐80	4630	968 (20.91%)		
Age‐group 2	30‐44	437	41 (9.38%)	37.071	3.64 × 10^−11^
	45‐80	4276	930 (21.75%)		
Age‐group 3	30‐49	900	104 (11.56%)	55.66	2.89 × 10^−15^
	50‐80	3813	867 (22.74%)		

**Table 5:** The numbers of advanced adenomas at different age‐groups

	Age‐groups	Cases of colonoscopy	Cases of advanced adenomas	*x* ^2^	*P* values
Age‐group 1	30‐39	83	2 (2.41%)	5.22	0.009
	40‐80	4630	460 (9.94%)		
Age‐group 2	30‐44	437	16 (3.66%)	20.55	3.73 × 10^−7^
	45‐80	4276	446 (10.43%)		
Age‐group 3	30‐49	900	47 (5.22%)	26.397	3.14 × 10^−8^
	50‐80	3813	415 (10.88%)		

For the anatomical position of advanced adenomas, the percentages of ascending colon and cecum, hepatic flexure, transverse colon, splenic flexure, descending colon, sigmoid colon, and rectal advanced were 17.96% (83 cases), 5.19% (24 cases), 12.77% (59 cases), 1.30% (6 cases), 13.64% (63 cases), 33.77% (135 cases), and 15.37% (71 cases), respectively (Table 3).

As for the size of adenomas, 269 cases were diminutive adenomas (<5 mm), 422 were small adenomas (5‐9 mm), and 280 were large adenomas (≥10 mm).

## 4 DISCUSSION

CRC is the third most commonly diagnosed cancer in men and the second in women worldwide and the fourth cause of cancer death worldwide. It accounts for over 9% of all cancer incidences, with an estimated 1.4 million cases occurring in 2012.^32^

The global burden of colorectal cancer (CRC) is expected to increase by 60% to more than 2.2 million new cases and 1.1 million deaths by 2030.The distribution of CRC varies widely across the world, with almost 55% of the cases occurring in more developed countries, especially in high‐income countries,^33^, ^34^ such as Australia, New Zealand, Europe, and Northern America. While the lowest incidence rates are in Africa, South‐Central Asia, and Central America, especially in Western Africa.

For twodecades, the CRC incidence rates have declined about 2% to 3% per year over the last 15 years in the United States, which is primarily associated with the increase in screening uptake and removal of precancerous adenomas.^35^, ^36^ In the United States, there were estimates that 136 830 people will be diagnosed with CRC and 50 310 people will die from it in 2014,^37^ while in 2015, approximately 132 700 individuals will be diagnosed with CRC and 49 700 patients will die from it.^38^ However, for recent years, the incidence rates of CRC are increasing in developing countries, particularly in Eastern Europe, Asia, and South America.^33^, ^39^ For some Asian regions, the CRC incidence rates have a rapid rise, and close to the rates reported in Western populations.^40^, ^41^

In China, the CRC incidence and mortality rates are increasing rapidly for two decades. There is obvious diversity of age, gender, and geographical difference in CRC incidence and mortality rates. The well‐developed provinces in China presented high incidence and mortality rates. Furthermore, CRC incidence and mortality rates were higher in urban areas than in rural regions. In 2010, there were about 274 841 new cases diagnosed with CRC (157 355 in males and 117 486 in females), with the crude incidence rate of 20.1/100 000, ranked the 6th in all cancer sites,^1^ and 132 110 cases died from it. In 2011, the new incidences were 310 244 cases (178 404 for males and 131 840 for females). The crude incidence rate was 23.03/100 000, made it fourth most common cancers in all cancer sites.^2^ In 2012, approximately 331 300 cases newly diagnosed with colorectal cancer and estimated 159 300 individuals died from it,^42^ While in 2015, it is estimated 376 300 new CRC cases diagnosed (males 215 700 and females 160 600), and 191 000 individuals died from CRC (males 111 100 and females 80 000).^43^ In Guangzhou, the incidence rates of CRC increased dramatically, from 22.2/10^5^ in 2000 to 34.0/10^5^ in 2011, higher than the average in China for the same time.^42^ ASRI increased from 20.5/10^5^ in 2000 to 23.2/10^5^ in 2011, and for male, the crude incidence increased from 23.4/10^5^ in 2000 to 37.4/10^5^ in 2011. Our screening data support this result.

The CRC incidence rates increase with age.^32^, ^43^, ^44^ In China, the majority of sporadic CRC cases were those aged 60‐74 years.^45^ Our results support those conclusions. In most programs, CRC screening in average‐risk populations commences around the age of 50‐60 years.^46^ The current guidelines recommend the first screening age of individuals at an average risk for CRC be started at the age of 50 years. While recently the incidence rate of CRC in adults aged below 50 years has increased greatly,^16^, ^47^, ^48^ Bailey et al predicted that by 2030, the incidence of CRC would increase by 27.7% to 46.0% in individuals aged 35 to 49 years.^16^ So Jung^49^ recommended colorectal cancer screening is necessary before 50 years of age.

Older age remains closely correlated with the incidence of adenomas,^29^, ^50^, ^51^ and the prevalence rate of adenomas increased sharply after 50 years old.^52^ Screening studies have revealed that 2.0% to 5.6% are found to have an advanced adenoma among subjects aged 40‐49 years old^8^, ^53^, ^54^ or large polyp. For both male and female, the risk of large polyps and advanced adenomas increases progressively with age, and beginning to accelerate at 50 years of age.^50^, ^55^ Many studies proved that adenoma prevalence was lower in female than in male,^50^ and our data indicated that for subjects over 40 years older, the detection rates of adenoma and advanced adenomas were 20.91% and 9.94%, respectively, and were higher than those younger.

For the adenoma distribution within the colon, Lieberman et al found that in persons younger than 60 years, more than half of CRC were located in the distal colon, whereas in persons aged 60 years and older, a slightly higher percentage of CRC were found in the proximal colon.^56^ Some studies reported a higher proportion of tumors occurring in the right colon.^57^, ^58^, ^59^ In the present study, the detection rate of advanced adenomas in the right colon was higher than reported before,^3^, ^58^, ^60^ and in the right colon, the detection rate of advanced adenomas was higher than that of CRC, suggested the incidence rate of CRC will increase in the right colon in the future. Therefore, colonoscopy is recommended and is the best choice for CRC screening.

In conclusion, our findings indicate that in Yuexiu District, Guangzhou, the detection rates of CRC, adenoma, and advanced adenoma are higher in male than in female. CRC screening is recommendation for the residents, especially for those over 40 years in male.

## CONFLICT OF INTERESTS

None declared.
